# Use of noninvasive ventilation in severe acute respiratory distress syndrome due to accidental chlorine inhalation: a case report

**DOI:** 10.5935/0103-507X.20170015

**Published:** 2017

**Authors:** Adriano Medina Matos, Rodrigo Ribeiro de Oliveira, Mauro Martins Lippi, Rodrigo Ryoji Takatani, Wilson de Oliveira Filho

**Affiliations:** 1 Programa de Residência em Medicina Intensiva, Hospital Universitário Getúlio Vargas, Universidade Federal do Amazonas - Manaus (AM), Brasil.; 2 Unidade de Terapia Intensiva, Hospital e Pronto-Socorro 28 de Agosto - Manaus (AM), Brasil.

**Keywords:** Respiratory distress syndrome, adult, Inhalation, Chlorine, Swimming pools, Case reports

## Abstract

Acute respiratory distress syndrome is characterized by diffuse inflammatory lung injury and is classified as mild, moderate, and severe. Clinically, hypoxemia, bilateral opacities in lung images, and decreased pulmonary compliance are observed. Sepsis is one of the most prevalent causes of this condition (30 - 50%). Among the direct causes of acute respiratory distress syndrome, chlorine inhalation is an uncommon cause, generating mucosal and airway irritation in most cases. We present a case of severe acute respiratory distress syndrome after accidental inhalation of chlorine in a swimming pool, with noninvasive ventilation used as a treatment with good response in this case. We classified severe acute respiratory distress syndrome based on an oxygen partial pressure/oxygen inspired fraction ratio <100, although the Berlin classification is limited in considering patients with severe hypoxemia managed exclusively with noninvasive ventilation. The failure rate of noninvasive ventilation in cases of acute respiratory distress syndrome is approximately 52% and is associated with higher mortality. The possible complications of using noninvasive positive-pressure mechanical ventilation in cases of acute respiratory distress syndrome include delays in orotracheal intubation, which is performed in cases of poor clinical condition and with high support pressure levels, and deep inspiratory efforts, generating high tidal volumes and excessive transpulmonary pressures, which contribute to ventilation-related lung injury. Despite these complications, some studies have shown a decrease in the rates of orotracheal intubation in patients with acute respiratory distress syndrome with low severity scores, hemodynamic stability, and the absence of other organ dysfunctions.

## Abstract (pt)

A síndrome do desconforto respiratório agudo é caracterizada por lesão pulmonar inflamatória difusa, classificada em leve, moderada e grave. Clinicamente observam-se hipoxemia, opacidades bilaterais na imagem pulmonar e diminuição da complacência pulmonar. A sepse está entre as causas mais prevalentes (30 - 50%). Dentre as causas diretas de síndrome do desconforto respiratório agudo, a inalação de cloro é uma causa incomum, gerando, na maior parte dos casos, irritação de mucosas e vias aéreas. Apresentamos um caso de síndrome do desconforto respiratório agudo grave após inalação acidental de cloro em piscina, sendo utilizada ventilação não invasiva como tratamento com boa resposta neste caso. Classificamos como síndrome do desconforto respiratório agudo grave baseado na relação pressão parcial de oxigênio/fração inspirada de oxigênio < 100, embora a classificação de Berlin seja limitada em considerar pacientes com hipoxemia grave manejados exclusivamente com ventilação não invasiva. A taxa de falha da ventilação não invasiva nos casos de síndrome do desconforto respiratório agudo está em torno de 52%, estando associada à maior mortalidade. As possíveis complicações do uso da ventilação mecânica não invasiva com pressão positiva na síndrome do desconforto respiratório agudo seriam o atraso para a intubação orotraqueal sendo a mesma realizada em uma condição clínica pior e um alto nível de pressões de suporte, somados a esforços inspiratórios profundos, gerando elevados volumes correntes e pressões transpulmonares excessivas, que contribuem para injúria pulmonar associada à ventilação. Apesar disto, alguns estudos mostraram diminuição nas taxas de intubação orotraqueal em pacientes com síndrome do desconforto respiratório Agudo com baixos escores de gravidade, estabilidade hemodinâmica e ausência de outras disfunções orgânicas.

## INTRODUCTION

Acute respiratory distress syndrome (ARDS) is a type of acute and diffuse inflammatory lung injury that leads to increased pulmonary vascular permeability and lung weight and the loss of aerated lung tissue.

Clinically, hypoxemia and bilateral opacities in lung images, increased physiological dead space, and decreased pulmonary compliance are observed. The morphological characteristic of the acute phase is diffuse alveolar damage, i.e., edema, inflammation, hyaline membrane formation, and hemorrhage.^(1)^ The process develops acutely (usually within 72 hours of the precipitating event) and can lead to death despite the institution of maximum therapy.^(2)^

The use of invasive mechanical ventilation is necessary in most cases of ARDS. The risks and benefits of noninvasive ventilation in ARDS are not yet defined, and the existing evidence does not support its routine use except for cases of mild ARDS without other organ dysfunctions.^(3)^ Multiple risk factors for ARDS have been identified, with sepsis having the highest prevalence (30 - 50%).^(2)^ ARDS is divided into direct and indirect causes.^(2)^ Among direct causes, chlorine inhalation injury is uncommon and rarely leads to ARDS.

According to the Berlin classification, severe ARDS requires early management with invasive mechanical ventilation.^(4)^ However, in the case reported here, we classified severe ARDS as an oxygen partial pressure/oxygen inspired fraction ratio (PaO_2_/FiO_2_) < 100 caused by accidental inhalation of chlorine during swimming pool cleaning, although the Berlin classification is limited in classifying patients with severe hypoxemia exclusively managed with noninvasive ventilation.

## CASE REPORT

A 55-year-old man accidentally inhaled a chlorine cloud when cleaning the swimming pool at his home, evolving to a clinical picture of mild dyspnea, cough with mucoid sputum, and epigastric pain. He sought emergency care (after 30 minutes), where he was initially evaluated for respiratory symptoms and received venous hydration, bronchodilators, and oxygen therapy. However, he presented progressive clinical worsening over a 3-hour period, with increased expectoration, arterial oxygen saturation (SpO_2_) (from 95% to 60%), and cyanosis and was referred to the reference emergency room, where he was seen in the emergency department. At that time, he already had signs of acute respiratory failure, associated with intense burning chest pain and cough with blood-tinged sputum. There was no history of smoking, respiratory diseases, and other comorbidities.

At admission, the patient was afebrile, tachycardic (heart rate 110bpm), and tachypneic (respiratory rate 34bpm), with a blood pressure of 134/82mmHg and an SpO_2_ of 86% during oxygen macronebulization at 10L/minute. Chest expandability was decreased due to pain, and respiratory auscultation detected the presence of generalized decreased vesicular murmur and crackling rales mainly on the lung bases. No other changes were detected on physical examination. Noninvasive ventilation with 60% FiO_2_, 7cmH_2_O support pressure (SP), and 10cmH_2_O positive end expiratory pressure (PEEP) was started.

**Figure 1:**
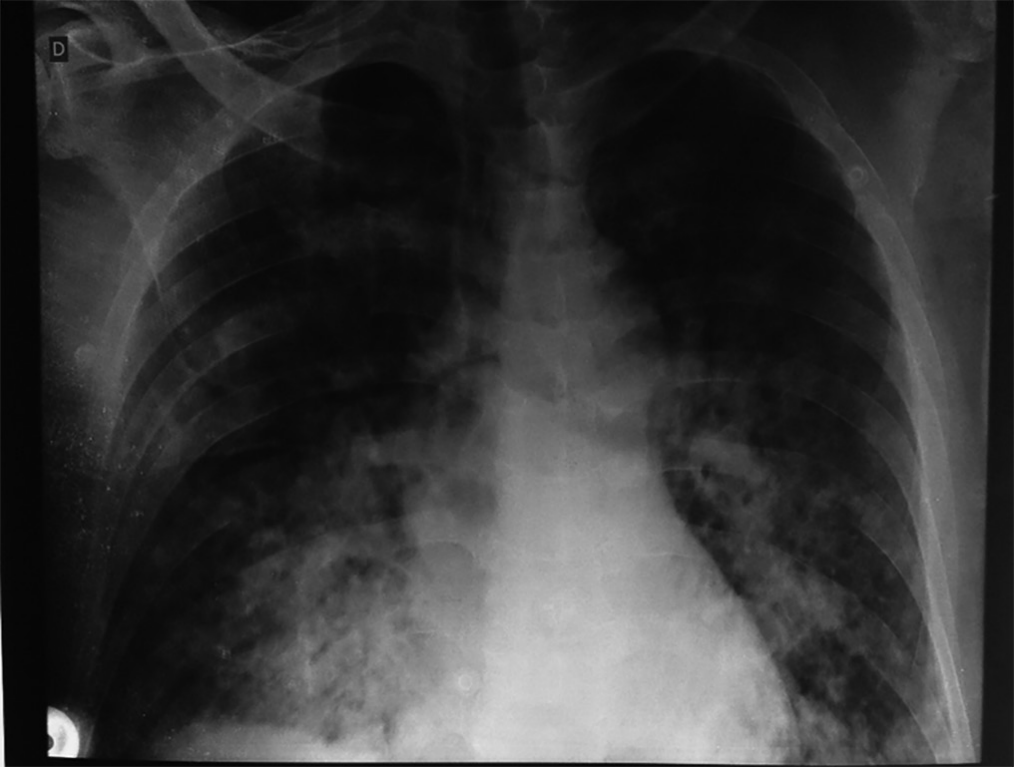
Initial chest X-ray.

**Figure 2:**
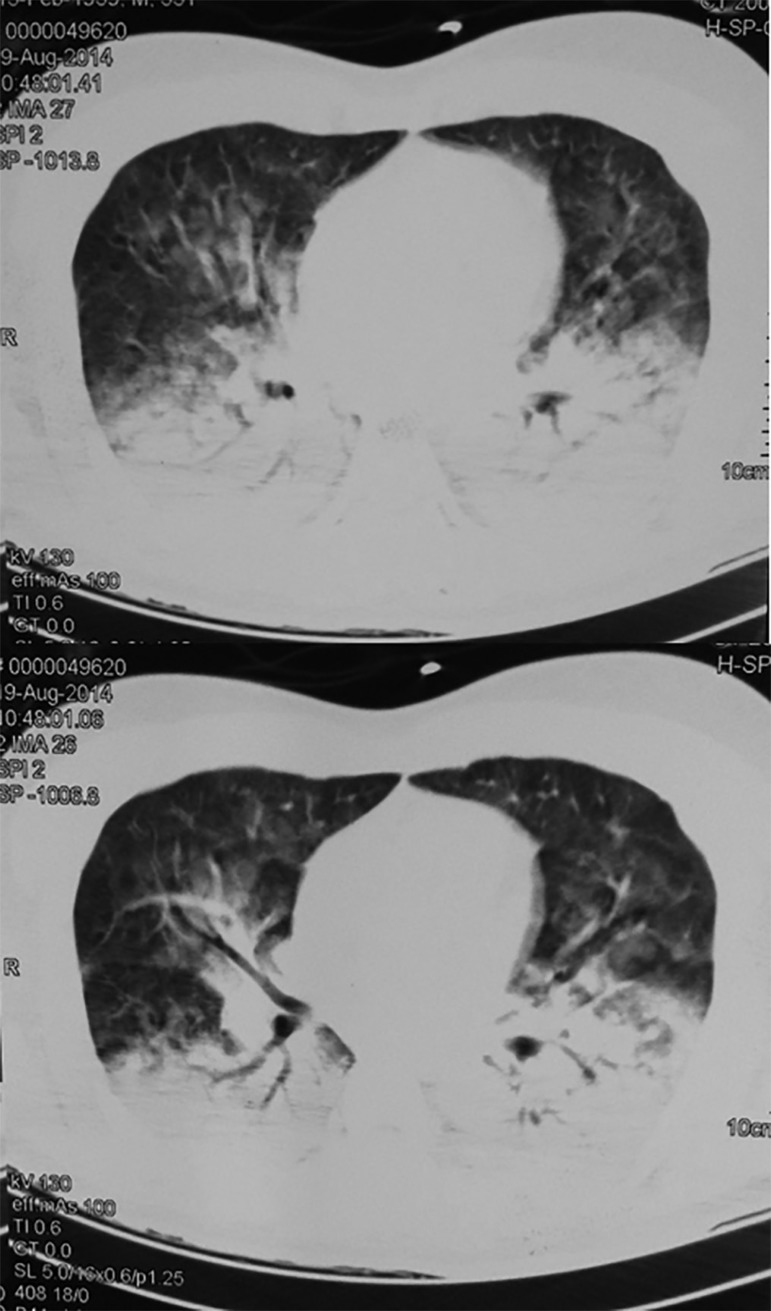
Initial chest computed tomography.

Arterial blood gas analysis revealed a pH of 7.41, a partial carbon dioxide pressure (PaCO_2_) of 39.1mmHg, a partial oxygen pressure (PaO_2_) of 59.5mmHg, an oxygen saturation (SO_2_) of 87.4%, a bicarbonate level of 29mEq/L and a base excess of +0.7mmol/L. The patient had leukocytosis (leukocyte count of 27,840 thousand/mm^3^ with 94% segmented). Electrolytes, coagulation tests, and liver and canalicular enzymes were normal. Chest X-ray revealed bilateral alveolar infiltrate in the lower third (Figure 1). Chest tomography showed consolidation in the posterior region of the lower lobes and discrete in the upper lobes, associated with areas of diffuse ground-glass opacity in the upper lobes, in addition to an increase in the diameter of the pulmonary arterial trunk (3.8cm), indicating hypertension of the same (Figure 2).

**Figure 3:**
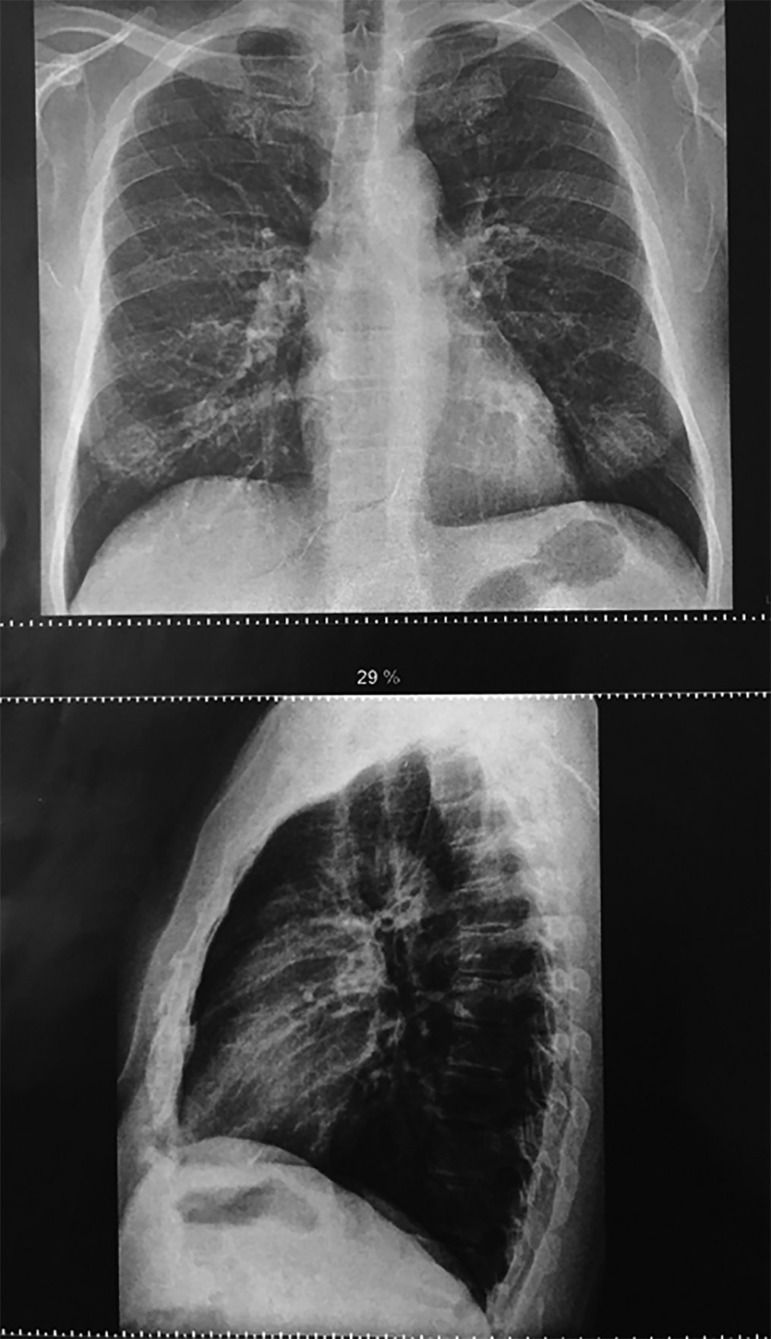
Chest X-ray: outpatient follow-up.

In the intensive care unit, antibiotic therapy with cefepime (2g every 8 hours), intravenous corticosteroid (methylprednisolone 125mg every 6 hours), and nebulization with a beta-agonist and an anticholinergic were started, with progressive clinical improvement and noninvasive mechanical ventilation (NIV) maintained intermittently for 4 days. A Dixtal^®^ 3010 mechanical ventilator with a full face mask interface was used in SP mode, using SP for a target tidal volume of approximately 6 to 8mL/kg predicted weight and a PEEP ranging from 5 to 10cmH_2_O, in addition to an FiO_2_ required to maintain an oxygen saturation above 88%. On the first day, the patient remained on a zero diet and remained on NIV for 24 hours; the PaO_2_/FiO_2_ ratio was 99. On the second day, pauses were taken for oral feeding; the PaO_2_/FiO_2_ ratio was 150. On the third day, most NIV shifts were maintained but with larger pauses; the PaO_2_/FiO_2_ ratio was 230. On the fourth day, NIV was performed intermittently; the PaO_2_/FiO_2_ ratio was 350. On the fifth day, the patient was kept on oxygen macronebulization. On the sixth day and following until hospital discharge, which occurred on the seventh day, the patient was spontaneously ventilated with room air. He remained hemodynamically stable throughout his hospital stay, without organ dysfunctions. He was discharged on the seventh day of hospitalization and was given an oral corticosteroid and a bronchodilator. In his outpatient follow-up, he underwent a chest X-ray, showing dense striae in the right upper lobe (Figure 3), with no other alterations. Spirometry was also performed but did not show alterations.

## DISCUSSION

Chlorine is a widely used industrial chemical and one of the ten most produced chemicals (by gross weight), being used for the production of plastics (28%), paper and cellulose (14%), solvents used in metalworking, dry cleaning and electronic cleaning (18%), water purification (5%), and in other chemicals, including pharmaceuticals (35%).^(5)^ Exposure to chlorine at toxic levels is often accidental and occurs during transport, in industrial exposures, or due improper use of cleaning products, such as in swimming pools. Because exposure to high levels of chlorine is always unintended, data on doses in these exposures are often not available. Likewise and for similar reasons, victims of chlorine exposure are treated empirically or inconsistently. The treatments evaluated were from uncontrolled studies, and exposure and treatment reports are anecdotal.^(6)^

The extent and severity of injuries caused by chlorine exposure depend on the duration of exposure, the gas concentration, the individual's susceptibility, the water content of the exposed tissues, and the minute ventilation of the exposed person.^(7)^

There are few reported cases of severe ARDS caused by chlorine inhalation in swimming pools. The most common manifestations in this type of exposure are mucosal and airway irritation, and almost all exposed individuals survived without sequelae. To our knowledge, there are three cases of ARDS in chlorine accidents in swimming pools, one of which died within a few hours. There has also been a case of diffuse bronchiolitis caused by chlorine inhalation associated with a swimming pool (Table 1).^(7-14)^

**Table 1:** Reported cases of exposure to chlorine in swimming pools

Author	Patients (N)	Manifestations
Babu et al.^(7)^	1	Acute respiratory distress syndrome
Decker and Kock^(8)^	1	Chest tightness and throat irritation
Decker^(9)^	41	Chest discomfort and nasal/throat discomfort
Martinez and Long^(10)^	2	Acute respiratory distress syndrome
Sexton and Pronchik^(11)^	13	Mucosal irritation, dyspnea, and wheezing
Kilburn et al.^(12)^	4	Not specified
Agabiti et al.^(13)^	182	Mucosal irritation, dyspnea, and wheezing
Parimon et al.^(14)^	1	Mucosal irritation, dyspnea, wheezing, and diffuse bronchiolitis

Chlorine exposure and accidental inhalation can cause a wide range of respiratory injuries, ranging from nasal irritation to pulmonary edema.

In addition to chlorine gas, other forms of chlorine are involved in airway toxicity, including hypochlorous acid, hydrochloric acid and chloramine. As chlorine gas has moderate solubility in water, it forms hypochlorous and hydrochloric acids when in contact with the moist surfaces of the airways. Although the exact mechanism of epithelial damage is not fully understood, oxidative injury is certainly involved, with chlorine gas (Cl_2_) combining with reactive oxygen species and other airway fluids to form a variety of highly reactive oxidants.

Direct injury to the epithelium can immediately initiate exposure to chlorine gas, while in indirect injury, inflammatory cells are activated and then migrate, with the subsequent release of oxidizing agents and proteolytic enzymes. Repair of the chlorine-induced epithelial injury may or may not occur, with reported cases of subepithelial fibrosis, mucosal hyperplasia, and non-specific bronchial hyperresponsiveness after recovery from a chlorine injury.

The equations related to the formation of hydrochloric acid, hypochlorous acid, oxygen and nitrogen compounds, which are present in scenarios of epithelial injury due to acute chlorine inhalation, can be summarized as follows:^(6)^

$$\begin{array}{c} {\mathrm{Cl}}_{2}+H_{2}O↔\mathrm{HCl}+\mathrm{HOCl}\\ 2\mathrm{HOCl}↔2\mathrm{HCl}+O_{2}\\ \mathrm{HOCl}+{\mathrm{NO}}_{2}\to \;\mathrm{reactive}\;\mathrm{nitrogen}\;\mathrm{species}\;(\mathrm{Cl}-\mathrm{ONO},\mathrm{Cl}-{\mathrm{NO}}_{2})\to \mathrm{Tyro}\sin e\to 3\mathrm{NT}\\ \mathrm{HOCL}+O_{2}\to \;\mathrm{hydroxyl}\;\mathrm{radicals}\;\mathrm{source}\;\left({\mathrm{OH}}^{-}\right)\\ O+2{\mathrm{NO}}^{-}\to 2{\mathrm{NO}}_{2}\\ O_{2}+\mathrm{NO}\to {\mathrm{ONOO}}^{-} \end{array}$$

In our case, the patient developed severe ARDS within a few hours and was treated with positive-pressure NIV. The use of NIV in this context is discouraged and controversial.

According to the Brazilian Guidelines on Mechanical Ventilation - 2013,^(15)^ NIV is recommended if there are no contraindications in patients with inability to maintain spontaneous ventilation (minute-volume > 4lpm, PaCO_2_ < 50mmHg and pH > 7.25), and NIV should be initiated with two pressure levels, with sufficient inspiratory pressure to maintain adequate ventilation to prevent the progression of muscle fatigue and/or respiratory arrest. NIV used for acute exacerbations of chronic obstructive pulmonary disease and acute cardiogenic lung edema decreases the need for endotracheal intubation and hospital mortality.

The Brazilian Guidelines on Mechanical Ventilation - 2013 suggest the use of NIV in mild cases of ARDS, observing the success targets of 0.5 to 2 hours (decrease in respiratory rate, increase in tidal volume, improvement in consciousness level, decrease or cessation of accessory muscle use, increase in PaO_2_ and/or SpO_2_ and decrease in PaCO_2_ without significant abdominal distention). In severe ARDS, the recommendation is to avoid using NIV because of the high rate of respiratory failure and the need for endotracheal intubation, especially in patients with PaO_2_/FiO_2_ < 140 and Simplified Acute Physiology Score (SAPS) II > 35.^(15)^

Nonetheless, the use of NIV for patients with ARDS remains unclear. In these patients, NIV failure is strongly predicted in patients with circulatory shock, metabolic acidosis, and elevated disease severity scores.^(16)^ A meta-analysis performed between 1995 and 2009 with 540 patients resulted in almost 50% failure of NIV in patients with ARDS.^(17)^ The possible complications of the use of NIV in ARDS include delayed orotracheal intubation, which is performed in poor clinical conditions and with high SP levels, along with deep inspiratory efforts, generating high tidal volumes and excessive transpulmonary pressures and contributing to ventilatory lung injury.^(18)^

The use of NIV as the first therapy in ARDS cases in centers experienced with this mode can prevent orotracheal intubation in 54% of patients, with SAPS II > 34 and the inability to increase PaO_2_/FiO_2_ after 1 hour being predictors of failure.^(8)^ Another retrospective study determined that in patients with acute alveolar injury treated with NIV, predictors for the need for orotracheal intubation include Acute Physiology and Chronic Health Disease Classification System II (APACHE II) score greater than 17 and respiratory rate greater than 25bpm after 1 hour of NIV.^(19)^ Recently, a case report of severe ARDS caused by H1N1 pneumonia was reported in a patient who, similarly to ours, had no other organ dysfunctions and was managed with successful NIV.^(20)^

## CONCLUSION

The use of noninvasive mechanical ventilation in cases of severe acute respiratory distress syndrome is still uncertain and currently discouraged. However, as in the case presented, noninvasive mechanical ventilation may be used in selected patients in cases of severe acute respiratory distress syndrome, especially in those with low severity scores, hemodynamic stability, absence of other organ dysfunctions, and improvement in the oxygen partial pressure/oxygen inspired fraction ratio in the first hour.
